# 

**Published:** 1910-03

**Authors:** 


### THE BRISTOL

### Medico-Chirurgical Journal.

A JOURNAL OF THE MEDICAL SCIENCES FOR THE WEST OF ENGLAND AND SOUTH WALES

*PUBLISHED UNDER THE AUSPICES OF*

*THE BRISTOL MEDICO-CHIRURGICAL SOCIETY.*

EDITOR:

R. SHINGLETON SMITH, M.D., B.Sc.,

WITH WHOM ARE ASSOCIATED

J. MICHELL CLARKE, M.A., M.D., J. LACY FIRTH, M.S., M.D., AND JAMES SWAIN, M.S., M.D.

ASSISTANT-EDITOR:

P. WATSON WILLIAMS, M.D.

EDITORIAL SECRETARY:

J. A. NIXON, B.A., M.B.

---

"Scire est nescire, nisi [illegible] Scire alius sciret."

---

LIBRARY

SURGEON GENERAL'S OFFICE

JUN. 4 1911

208561.

*VOL. XXVIII.*

BRISTOL: J. W. ARROWSMITH.
LONDON: J. & A. CHURCHILL, 7 GREAT MARLBOROUGH STREET.
1910.

WI
BR323

J. W. Arrowsmith Publisher Bristol.

### CONTENTS OF VOLUME XXVIII.

Page

Radium and some of its Physical and Therapeutic Properties. By James Mackenzie Davidson, M.B., C.M. .. .. .. 1

Diffuse Destructive Changes in the Liver Associated with Jaundice. By Carey Coombs, M.D., M.R.C.P. Lond. .. .. .. 17

Infection of the Urinary Tract with Bacillus Coli. By J. R. Charles, M.A., M.D. Cantab., M.R.C.P. .. .. .. .. .. 23

Hypnotism. By Hugh P. Costobadie, M.R.C.S., L.R.C.P... .. 33

Oriental Medicine—Stray Jottings. By V. B. Green-Armytage, I.M.S. .. .. .. .. .. .. .. .. .. 40

The Long Fox Lecture: The Development of the Human Skull. By Edward Fawcett, M.D. .. .. .. .. .. 97

Notes on Biliary and Intestinal Sand. By George Parker, M.D... 112

Notes on a Case of Oxycephaly. By G. Hely-Hutchinson Almond, M.B., M.A. Oxon. .. .. .. .. .. .. .. 118

Iodoform and Thyroidism. By A. Rendle Short, M.D., B.Sc., F.R.C.S. .. .. .. .. .. .. .. .. 122

A Case in which a Stone formed in the Urethra around a piece of Wood introduced into the Urethra twenty-six years before the Removal of the Stone. By Chas. A. Morton, F.R.C.S. .. 127

Bromoform Poisoning. By Rupert Waterhouse, M.D., M.R.C.P. Lond. .. .. .. .. .. .. .. .. .. 129

Post-Graduate Study. By J. M. Rattray, M.A., M.D. .. .. 193

Cases of Leukæmia treated by X-Rays. By J. Michell Clarke, M.A., M.D. Cantab., F.R.C.P. .. .. .. .. .. 208

A Brief Account of Splenectomy with a Case of Simple Hypertrophy of the Spleen treated by Operation. By James Swain, M.S., M.D. Lond., F.R.C.S... .. .. .. .. .. .. 223

CONTENTS.

| | Page |
| --- | --- |
| Radiographic Estimation of Simple Enlargement by means of Visible Shadows. By WILLIAM COTTON, M.A., M.D., D.P.H... | 226 |
| A Case of Anomalous Cutaneous Eruption showing a Bacillus morphologically corresponding with the Klebs-Lœffler, and clearing up under Diphtheritic Antitoxin. By W. KENNETH WILLS, M.A., M.B., B.C. Cantab. .. .. .. .. .. | 231 |
| The Medical Aspect of Boswell's *Life of Johnson*, with some Account of the Medical Men mentioned in that book. By BERTRAM M. H. ROGERS, M.D., B.Ch., B.A. Oxon... .. .. .. .. | 289 |
| Remarks on the Surgical Aspect of Glycosuria, and on Diabetic Gangrene, with the Record of Three Cases in which Amputation was performed for Gangrene in Diabetics under Spinal Anæsthesia. By CHARLES A. MORTON, F.R.C.S... .. .. .. .. | 311 |
| Hyoscine and the Mydriatic Alkaloids. By J. M. FORTESCUE-BRICKDALE, M.A., M.D. Oxon. .. .. .. .. .. | 317 |
| On the Excision of Strictures of the Urethra. By ERNEST W. HEY GROVES, M.S., F.R.C.S. .. .. .. .. .. .. | 325 |
| An Old-Time Syphilidiater. By HUBERT J. NORMAN, M.B., D.P.H. | 334 |
| PROGRESS OF THE MEDICAL SCIENCES .. .. 45, 140, 235, | 343 |
| REVIEWS OF BOOKS .. .. .. .. 56, 151, 247, | 357 |
| EDITORIAL NOTES .. .. .. .. .. 74, 172, 260, | 363 |
| NOTES ON PREPARATIONS FOR THE SICK .. 83, 180, 268, | 370 |
| THE LIBRARY OF THE BRISTOL MEDICO-CHIRURGICAL SOCIETY .. .. .. .. 85, 183, 274, | 374 |
| MEETINGS OF SOCIETIES.. .. .. .. .. 88, 185, | 376 |
| OBITUARY .. .. .. .. .. .. .. 93, 276, | 379 |
| LOCAL MEDICAL NOTES .. .. .. .. 94, 188, 286, | 380 |

### Bristol Medico-Chirurgical Society.

### President.

JAMES SWAIN, M.D., M.S. Lond.

### President-Elect.

B. M. H. ROGERS, M.A., M.D., B.Ch. Oxon.

### Committee.

*Ex-officio*
- J. MICHELL CLARKE, M.A., M.D. Cantab.
- R. SHINGLETON SMITH, M.D. Lond. (Editor of *Journal*).
- C. K. RUDGE, M.R.C.S. (Hon. Librarian).
- J. A. NIXON, B.A., M.B. Cantab. (Editorial Secretary).

*Elected.*

J. PAUL BUSH, C.M.G. } Ex-Presidents.
JAMES TAYLOR, M.R.C.S. }
P. WATSON WILLIAMS, M.D. Lond.
G. PARKER, M.A., M.D. Cantab.
I. WALKER HALL, M.D. Vict.
F. LACE, F.R.C.S.
J. LACY FIRTH, M.D., M.S. Lond.
C. A. MORTON, F.R.C.S.

### Honorary Secretary.

J. A. NIXON, B.A., M.B. Cantab.

Medical Practitioners wishing to join the Society are requested to communicate with the Hon. Secretary, Dr. NIXON, 18 West Mall, Clifton, Bristol. The Annual Subscription is 21/-.

---

*Extracts from Journal By-laws.*

4.—The *Journal* shall be delivered free to Subscribers and also to Members of the Society whose subscriptions are not in arrear.

6.—The Annual Subscription to the *Journal* for those who pay before the 1st of July shall be 5/-, post free, or 6/- if paid after that date.

---

Communications intended for insertion in the *Journal* should be sent to the Editor, Dr. SHINGLETON SMITH, Deepholm, Clifton Park, Clifton, Bristol.

Books for Review and Exchange Journals should be sent to the Assistant-Editor, *Bristol Medico-Chirurgical Journal*, Medical Library, The University, Bristol.

Communications referring to the delivery of the *Journal* should be sent to the Editorial Secretary, Dr. J. A. NIXON, 18 West Mall, Clifton, Bristol, to whom Subscriptions are to be paid *in advance*.

Cases for Binding Vols. I–XXVI are now ready, and may be obtained, price 7*d*. each (postage 2*d*. extra), upon application to J. W. ARROWSMITH, Quay Street, Bristol; or the *Journal* will be bound, including Case, for 1/3 per volume.

### Bristol Medico-Chirurgical Society.

---

### PAST PRESIDENTS.

1874—75 FREDERICK BRITTAN, M.D., B.A. Dub.
1875—76. ROBERT WILLIAM COE.
1876—77. SAMUEL MARTYN, M.D. Ed.
1877—78. AUGUSTIN PRICHARD, M.D. Berlin.
1878—79. HENRY EDWARD FRIPP, M.D. Würzburg.
1879—80. WILLIAM MICHELL CLARKE.
1880—81. JOSEPH GRIFFITHS SWAYNE, M.D. Lond.
1881—82. EDWARD LONG FOX, M.D. Oxon.
1882—83. JAMES GEORGE DAVEY, M.D. St. And.
1883—84. WILLIAM JOHNSTONE FYFFE, M.D., B.A. Dub.
1884—85. GEORGE FORSTER BURDER, M.D. Aberd.
1885—86. WILLIAM HENRY SPENCER, M.D., M.A. Cantab.
1886—87. FRANCIS POOLE LANSDOWN.
1887—88. LEMUEL MATTHEWS GRIFFITHS.
1888—89. ROBERT SHINGLETON SMITH, M.D., B.Sc. Lond.
1889—90. NELSON CONGREVE DOBSON.
1890—91. SAMUEL HENRY SWAYNE.
1891—92. FRANCIS RICHARDSON CROSS, M.B. Lond.
1892—93. EDWARD MARKHAM SKERRITT, M.D., B.S., B.A. Lond.
1893—94. JAMES GREIG SMITH, M.B., C.M., M.A. Aberd., F.R.S.E.
1894—95. ALFRED JAMES HARRISON, M.B. Lond.
1895—96. ARTHUR WILLIAM PRICHARD.
1896—97. ALFRED EDWARD AUST LAWRENCE, M.D., C.M. Aberd.
1897—98. JOHN EDWARD SHAW, M.B., C.M. Ed.
1898—99. ROBERT ROXBURGH, M.B. Ed.
1899—00. WILLIAM HENRY HARSANT.
1900—01. DAVID SAMUEL DAVIES, M.D. Lond.
1901—02. BARCLAY JOSIAH BARON, M.B., C.M. Ed.
1902—03. GEORGE MUNRO SMITH.
1903—04. JAMES PAUL BUSH, C.M.G.
1904—05. REGINALD EAGER, M.D. Lond.
1905—06. JOHN DACRE.
1906—07. JAMES TAYLOR.
1907—08. HENRY WALDO, M.D., C.M. Aberd
1908—09. J. MICHELL CLARKE, M.D. Cantab.
1909—10. J. SWAIN, M.D., M.S. Lond.

1910.

### LIST OF SUBSCRIBERS

TO THE

### Bristol Medico-Chirurgical Journal.

*Those marked * are Members of the Society.*

| | |
|---|---|
| ACKLAND, J. McKNO ... ... ... ... ... ... | 1 Barnfield Crescent, Exeter. |
| *ACKLAND, W. R. ... ... ... ... ... ... ... | 5 Rodney Place, Clifton. |
| ADYE, W. J. A. ... ... ... ... ... ... ... .. | Church House, Bradford, Wilts. |
| ALDOUS, GEORGE F.... ... ... ... ... ... ... | Charlton House, Mannamead, Plymouth |
| *ALEXANDER, D.A., M.B., Ch.B. Vict.... ... ... | 30 Berkeley Square, Clifton. |
| AMBROSE, J., M.B. C.M. Aberd. ... ... ... ... | 138 Fishponds Road, Eastville, Bristol. |
| *AUBREY, T., M.B. ... ... ... ... ... ... ... | Bitton, near Bristol. |
| AVELINE, H. T. S. ... ... ... ... ... ... ... | Somerset Asylum, Taunton. |

| | |
|---|---|
| *BALLANCE, H. STANLEY, M.D. Lond. ... ... | 4 Royal Terrace, Weston-super-Mare |
| BARBER, M. C. ... ... ... ... ... ... ... | Staple Hill, Bristol. |
| BARETTI, T. G. L'ENARDI ... ... ... ... .. | 49 Royal York Crescent, Clifton. |
| *BARKER, G. H., M.B., B.Sc. Lond. ... ... ... | 124 Redland Road, Bristol |
| *BARON, BARCLAY J., M.B., C.M. Ed.... ... ... | 16 Whiteladies Road, Clifton. |
| BASSET, WALTER ... ... ... ... ... ... ... | 24 Stow Hill, Newport, Mon. |
| BATTEN, W. SMITH... ... ... ... ... ... ... | West Hill, Calne. |
| BAYER CO., THE ... ... ... ... ... ... ... | 19 St. Dunstan's Hill, London, E.C. |
| *BEDDOE, JOHN, M.D. Ed., B.A. Lond., F.R.S. | The Chantry, Bradford-on-Avon. |
| *BENSON, J. ... ... ... ... ... ... ... | 11 The Circus, Bath. |
| *BERGIN, F. G. ... ... ... ... ... ... ... | 12 West Park, Clifton. |
| BERNARD, C. ... ... ... ... ... ... ... ... | 2 Spencer Terrace Fishponds. |

### LIST OF SUBSCRIBERS.

| | |
|---|---|
| *Berry, F. C., M.D., B.Ch., B.A. Dub. ... ... | Burnham, Somerset. |
| Berry, James, M.B., B.S. Lond. ... ... ... | 21 Wimpole Street, London, W. |
| Biamonti, Sebastiano ... ... ... ... ... ... ... | 27 Via Balbi, Genoa, Italy. |
| *Blacker, A. E.... ... ... ... ... ... ... ... ... | 20 Victoria Square, Clifton. |
| *Blacker, E., M.D. Durh. ... ... ... ... ... | 8 Meridian Road, Redland. |
| *Blackett, J. F., M.B., B.S. Lond. ... ... ... | Audley, Newcastle, Staffs. |
| *Blackley, F. J., M.D. Dub. ... ... ... ... | 2 Knowle Road, Bristol. |
| *Blagg, Arthur F., M.D. Durh.... ... ... ... | 28 Caledonia Place, Clifton. |
| Board, J. H. ... ... ... ... ... ... ... ... ... | 8 Caledonia Place, Clifton. |
| Bodman, Hervey, M.D. Lond. ... ... ... ... | 3 Whiteladies Road, Clifton. |
| *Boucher, F. T. ... ... ... ... ... ... ... ... | Hazlemere, Cotham Road, Bristol. |
| *Bowker, G. E., M.D. Edin.... ... ... ... ... | 11 North Parade, Bath. |
| Boyce, James G. ... ... ... ... ... ... ... ... | The Chipping, Wotton-under-Edge. |
| Boyd, Geo. S. J. ... ... ... ... ... ... ... ... | 19 St. George's Square, Stamford, Lincs. |
| *Brasher, Charles W. J. ... ... ... ... ... | 128 Cotham Brow, Bristol. |
| *Brew, R. H. ... ... ... ... ... ... ... ... ... | Chew Magna. |
| *Bristowe, H. C., M.D. Lond. ... ... ... ... | Wrington, Somerset. |
| Brown, G. Arthur ... ... ... ... ... ... ... | Tredegar. |
| Brown, William, M.D., C.M. Glasg. ... ... | Park View, Fishponds, Gloucestershire. |
| *Bullen, F. St. John ... ... ... ... ... ... | 12 Pembroke Road, Clifton. |
| *Bunbury, E. G. ... .. ... ... ... ... ... ... | 13 Dowry Square, Hotwells. |
| Burroughs, E. F. H. ... ... ... .. ... ... | 27 Burlington Road, Redland, Bristol. |
| *Bush, J. Paul, C.M.G. ... ... ... ... ... ... | Vyvyan House, Clifton Park, Clifton. |

| | |
|---|---|
| *Cadman, E. S. R., M.D., C.M. Edin. ... ... | 87 St. Michael's Hill, Bristol. |
| *Carling, A., M.A., M.B., B.C. Cantab. ... ... | 12 Great George Street, Bristol. |
| *Carter, T. M., M.D. Durh. ... ... ... ... | Burnley, Westbury-on-Trym. |
| *Carwardine, Thomas, M.B., M.S. Lond. ... | 16 Victoria Square, Clifton. |
| *Cave, Edward J., M.D. Lond. ... ... ... ... | 20 Circus, Bath. |
| *Charles, J. R., M.A., M.D., B.C. Cantab. ... | 12 Victoria Square, Clifton. |
| *Chitty, H., M.S. Lond. ... ... ... ... ... | Royal Infirmary, Bristol. |
| Chute, Henry M. ... ... ... ... ... ... ... ... | King William's Town, S. Africa. |
| *Clarke, C., M.B., B.S. Lond. ... ... ... ... | 17 Elmdale Road, Clifton. |
| *Clarke, John Michell, M.D., M.A. Cantab. | 28 Pembroke Road, Clifton. |
| Cobbledick, A. S., M.D., B.S. Lond. ... ... | 402 Brixton Road, London, S.W. |
| *Coleridge, Alfred, M.B., B.S. Lond. ... ... | 154 Coronation Road, Bristol. |
| *Collinson, T. A. ... ... ... ... ... ... ... ... | 1 Apsley Villas, Kingsdown, Bristol. |
| *Connellan, E. V. ... ... ... ... ... ... | 218 Caerleon Road, Newport, Mon. |
| Connellan, P. S. ... ... ... ... ... ... ... ... | Gordon Hosp., Vauxhall Bridge Road, London, S.W. |
| *Cook, Henri, M.D., C.M. Aberd. ... ... ... | 1 Dean Lane, Southville, Bristol. |
| *Coombs, Carey, M.D. Lond. ... ... ... ... | 74 St. Paul's Road, Clifton. |
| *Corfield, C. .. ... .. ... ... ... ... ... ... | 188 Wells Road, Knowle. |
| Cossham, W. R., M.D., C.M. Aberd.... ... ... | Cirencester. |
| Cotton, William, M.B., C.M., M.A. Ed. ... | 231 Gloucester Road, Bristol. |
| Coulter, R. J., M.B. Dub. ... ... ... ... ... | 11 Clytha Park Road, Newport, Mon. |
| Cridland, A. B. ... ... ... ... ... ... ... ... | 52 Waterloo Road, Wolverhampton. |
| *Cross, F. Richardson, M.B. Lond.... ... ... | Worcester House, Clifton. |
| *Crossman, F. W. ... ... ... ... ... . ... | Hambrook, Gloucestershire. |
| *Crouch, C. Percival, M.B. Lond. ... ... ... | 1 Royal Terrace, Weston-super-Mare. |

| | |
|---|---|
| *Dacre, John ... ... ... ... ... ... ... ... | 14 Eaton Crescent, Clifton. |
| *Davies, D. S., M.D. Lond., D P.H. Cantab. ... | 60 Oakfield Road, Clifton. |
| *Davies, G. A. ... ... ... ... ... ... ... ... | 11 Alexandra Road, Clifton. |

| | |
|---|---|
| DENING, EDWIN ... ... ... ... ... ... ... | Manor House, Stow-on-the-Wold. |
| DEVINE, H. ... ... ... ... ... ... ... ... | Long Grove, Epsom. |
| DICKIE, JAMES STEEL, M.D., C.M. Aberd. ... | Boscombe Park, Bournemouth. |
| *DICKINSON, W. G., M.D., Durh. ... ... ... | Wood Hill, Adelaide Terr., Portishead. |
| *DOBSON, NELSON C. ... ... ... ... ... ... | 16 College Road, Clifton. |
| *DOWLING, E. A. G. ... ... ... ... ... ... | 5 Rodney Place, Clifton. |
| DUNBAR, ELIZA L. WALKER, M.D. Zürich ... | 9 Oakfield Road, Clifton. |
| DYMOKE, F. ... ... ... ... ... ... ... ... | 21 Cotham Road, Bristol. |

| | |
|---|---|
| EADON, W. F. BAILEY ... ... ... ... ... ... | Hambrook, Gloucestershire. |
| *EAGER, REGINALD, M.D. Lond. ... ... ... ... | Northwoods, Winterbourne. |
| *EDGEWORTH, F. H., M.B., B.C., B.A. Cantab., D.Sc. Lond. ... ... ... ... ... ... ... ... | 35 Pembroke Road, Clifton. |
| EDWARDS, E. S. ... ... ... ... ... ... ... | Empingham, Stamford. |
| ELDER, WILLIAM ... ... ... ... ... ... ... | 4 Trelawney Road, Bristol. |
| ELLIOT BROS. LTD. ... ... ... ... ... ... | *Australian Pharmaceutical Notes & News*, O'Connell Street, Sydney, N.S.W. |
| *ELLIOTT, CHRISTOPHER, M.D., B.A. Dub. ... | 3 Beaufort Road, Clifton. |
| ELLIOTT, F. PERCY, M.B., C.M. Aberd. ... ... | 113 Grove Road, Walthamstow, London, N.E. |
| *ELLIOTT, W. H. A., M.B., B.S. Lond. ... ... | Rockfort, Arley Hill, Bristol. |
| *EMRYS-ROBERTS, E., M.D. Liverpl. ... ... | 5 Buckingham Place, Clifton. |
| EVANS, T. G. C. ... ... ... ... ... ... ... | Budleigh Salterton, Devon. |
| *EVERY-CLAYTON, L. E. V., M.D. Lond. ... ... | Rosslyn, Clevedon, Somerset. |

| | |
|---|---|
| *FELLS, A., M.B., C.M. Edin. ... ... ... ... | 107 Ashley Road, Bristol. |
| *FERGUSON, R. S , B.A. Durh., M.B. C.M.Edin. | Elm Grove, Calne, Wilts. |
| *FIRTH, J. L., M.D., M.S. Lond. ... ... ... ... | 8 Victoria Square, Clifton. |
| *FLEMMING, A. L. ... ... ... ... ... ... ... | 34 Alma Road, Clifton. |
| *FLEMMING, C. E. S. ... ... ... ... ... ... | Manvers House, Bradford-on-Avon. |
| *FLETCHER, J., M.D., C.M., D.P.H. Aberd. ... | Ham Green, Somerset. |
| *FORTESCUE-BRICKDALE, J. M., M.A., M.D., B.Ch. Oxon. ... ... ... ... ... ... ... | 52 Pembroke Road, Clifton. |
| FORTY, D. H. ... ... ... ... ... ... ... ... | Wotton-under-Edge. |
| FOSS, E. V. ... ... ... ... ... ... ... ... | Clouds Hill House, St. George, Bristol. |
| FOX, F. F. ... ... ... ... ... ... ... ... | Yate House, Yate. |
| *FRASER, FORBES ... ... ... ... ... ... ... | 2 The Circus, Bath. |
| FREEMAN, J., M.D. Brux. ... ... ... ... ... | Glencairn Villa, Queen's Road, Clifton. |

| | |
|---|---|
| GARLAND, E. B., M.B., C.M. Edin. ... ... ... | 114*a* Hampton Road, Redland. |
| GEE, C. A. H. ... ... ... ... ... ... ... ... | Evercreech, Somerset. |
| GENGE, T. T. ... ... ... ... ... ... ... ... | 21 Whiteladies Road, Bristol. |
| *GERRISH, D. S.... ... ... ... ... ... ... ... | 322 Church Road, St. George, Bristol. |
| *GIBBS, A. N. GODBY ... ... ... ... ... ... | 52 Whiteladies Road, Clifton. |

### LIST OF SUBSCRIBERS.

Goss, T. Biddulph... ... ... ... ... ... ... 1 The Circus, Bath.

*Gratte, C. Brooke ... ... ... ... ... ... 19 Gold Tops, Newport, Mon.

*Green, T. A., M.D., C.M. Edin. ... ... ... 33*a* Whiteladies Road, Clifton.

Green-Armytage, Lt. V. B., R.A.M.C. ... c/o King & Co., Bombay.

*Greer, W. J. ... ... ... ... ... ... ... ... 19 Gold Tops, Newport, Mon.

*Grey, Harry, M.D:, C.M.Edin. ... ... ... 492 Fishponds Road, Bristol.

Griffiths, Cornelius A. ... ... ... ... ... 6 Windsor Place, Cardiff.

*Griffiths, J. S. ... ... ... ... ... ... ... 20 Redland Park, Bristol.

*Griffiths, L. M. ... ... ... ... ... ... ... 11 Pembroke Road, Clifton.

*Groves, E. W. H., M.B., B.Sc. Lond. ... ... 16 Richmond Hill, Clifton.

*Hailes, Clements D. G., M.D., C.M. Ed. ... 27 Alma Road, Clifton.

*Hall, E. G., M.B. Lond. ... ... ... ... ... 53 Hampton Road, Redland, Bristol.

*Hall, I. Walker, M.D., Ch.B.Vict. ... ... 9 Royal Park, Clifton.

Handfield-Jones, Montagu, M.D. Lond. ... 35 Cavendish Square, London, W.

*Harris, H. Elwin, B.A., M.B. Cantab. ... ... 13 Lansdown Place, Clifton.

*Harsant, W. H. ... ... ... ... ... ... ... Tower House, Clifton.

Hartnell, E. B. ... ... ... ... ... ... ... 13 Church Street, Bridgwater.

*Harvey, Alfred, M.B. Lond. ... ... ... ... Westbury-on-Trym.

*Hawes, C. S. ... ... ... ... ... ... ... ... Buenos Ayres, Argentina.

*Hawes, I. H., M.B., B.S. Durh. ... ... ... Wick, Glos.

Hayman, Alfred G. ... ... ... ... ... ... Hapsford House, near Frome.

*Hayman, C. A., M.D. St. And. ... ... ... ... Kingston Villa, Richmond Hill, Clifton

*Herapath, C. E. K., M.B. Lond. ... ... ... 12 Brunswick Square, Bristol.

*Herapath, C. K. C. ... ... ... ... ... ... Cotham Park, Bristol.

Hill, Hedley ... ... ... ... ... ... ... ... 1 Whiteladies Road, Clifton.

*Hill, Walter J. ... ... ... ... ... ... ... The Brow, Clevedon.

*Home, W. E., M.D., Edin., Fleet-Surgeon R.N. H.M.S. *Dædalus*, Bristol.

Hospital, Bristol General (*Sec.*, W. Thwaites).

Hospital, Taunton and Somerset (*House-Surgeon*, W. Drake).

Huggard, W. R., M.D., M.Ch., M.A. Roy. Univ. ... ... ... ... ... ... ... ... ... Davos-Platz, Switzerland.

*Iles, A. E., M.R.C.S. ... ... ... ... ... ... 1 Cotham Lawn, Bristol.

Imlay, G. A., M.B., C.M. Aberd. ... ... ... 1 Cotham Park, Bristol.

Infirmary, Bristol Royal (*Secretary*, W. E. Budgett).

Ingle, C. Durban ... ... ... ... ... ... ... Somerton, Somerset.

*Irwin, S., M.B., B.Ch., B.A.O., R.U.I. ... ... Olveston, Glos.

*James, C. W. W. ... ... ... ... ... ... ... 246 Wells Road, Knowle.

Jamison, A., M.B., B.Ch., M.A. R.U.I. ... ... Ardenvohr, Woodstock Road, Belfast.

Jones, Evan ... ... ... ... ... ... ... ... Ty-mawr, Aberdare.

*Jones, E. J. Trevor, M.D. Brux. ... ... ... Aberdare, Glamorganshire.

*Jones, J. Ellington ... ... ... ... ... ... 16 Richmond Park Road, Clifton.

Jones, H. Macnaughton, M.D., M.Ch. R.U.I. ... 131 Harley Street, London, W.
*Jones, R. Llewellyn, M.B. Lond. ... ... ... 40 Gay Street, Bath.
Joseph, A. Hill, M.D. Lond. ... ... ... ... Bexhill-on-Sea.

*Keir, I. C., M.B., Ch.B. Ed. ... ... ... ... The Limes, Melksham.
*Kemm, F. St. J.... ... ... ... ... ... ... ... Worle.
*Kemp, C. G., M.B., B.S. Durh. ... ... ... ... Oakhurst, St. Helens Park, Hastings.
*Kyle, H. G., M.B., B.Ch. Oxon. ... ... ... 31 Westbury Road, Westbury-on-Trym.

*Lace, F. ... ... ... ... ... ... ... ... ... ... 5 Gay Street, Bath.
*Lansdown, R. G. P., M.B., B.S. Dur. ... ... 39 Oakfield Road, Clifton.
*Lavington, C. C., M.B., B.S. Dur. ... ... 1 Burlington Road, Redland.
Lendon, A. A., M.D. Lond. ... ... ... ... North Terrace, Adelaide, S. Australia.
Le Soudier, H. ... ... ... ... ... ... ... 174 et 176 Boulevard St. Germain, Paris.
*Lees, E. Leonard, M.D., C.M. Ed. ... ... ... 1 Chesterfield Place, Clifton.
Leigh, W. W. ... ... ... ... ... ... ... ... ... Treharris, Glamorganshire.
*Leonard, R. C., M.D. Ed. ... ... ... ... ... 134 Coronation Road, Bristol.
*Lindsay, J., M.D. Ed. ... ... ... ... ... ... 24 Bennett Street, Bath.
*Lucas, J. J. S., M.D. Lond. ... ... ... ... ... 186 Wells Road, Totterdown.
Lucy, Reginald H. ... ... ... ... ... ... 9 The Crescent, Plymouth.

*McCrea, P. W., M.D., B.Ch., B.A.O., B.A. Dub. ... ... ... ... ... ... ... ... ... ... 19 Portland Square, Bristol.
MacDonald, P. W., M.D., C.M. Aberd. ... ... Herrison, Dorchester.
Mackinnon, Charles, M.B., C.M., M.A. Glasg. Davaar, Cirencester.
Maclean, Ewen J., M.D., C.M. Ed.... ... ... 12 Park Place, Cardiff.
Maclean, J. Campbell, M.B., C.M. Ed.... ... Swindon House, Swindon, Wiltshire.
*Macleod, D., M.B., B.C. Ed. ... ... ... ... 84 Redland Road, Bristol.
*MacWatters, J. C. .. ... ... ... ... ... ... Almondsbury.
Marsh, O. E. Bulwer ... ... ... ... ... ... Parkdale, Newport, Mon.
Marshall, Arthur L., M.B., B.A. Cantab ... 206 Upper Clapton Road, London, N.E.
*Martin, E. F., M.B. Ed. ... ... ... ... ... 7 Royal Terrace, Weston-super-Mare.
*Martin, R C. ... ... ... ... ... ... ... ... 3 Clifton Vale, Clifton.
*Martyn, G. J. King, M.D., B.C., B.A. Cantab. ... ... ... ... ... ... ... ... ... 12 Gay Street, Bath.
Mason, S. B. ... ... ... ... ... ... ... ... 12 Park Terrace, Pontypool.
Mason, William ... ... ... ... ... ... ... Sedgemoor Cottage, St. Austell, Cornwall.
Matthews, Charles E., M.D. Dur. ... ... St. Aubyn's Park, Tiverton.
Meredith, John, M.D. Ed. ... ... ... ... ... Wellington, Somerset.
Miall, C. L. ... ... ... ... ... ... ... ... ... Elm Hayes, Paulton, near Bristol.
*Mole, Harold F. ... ... ... ... ... ... ... 19 Mortimer Road, Clifton, Bristol.
Monckton, William ... ... ... ... ... ... Portishead.

| | |
|---|---|
| *Moore, C. A., M.B., M.S. Lond. ... ... ... ... | General Hospital, Bristol. |
| *Moore, L. A. ... ... ... ... ... ... ... ... | 45 Ashley Road, Bristol. |
| Moores, De la Hey ... ... ... ... .. ... | 6 West Mall, Clifton. |
| *Morgan, T. Whitworth S. ... ... ... ... | The Laurels, Timsbury, Bath. |
| *Morton, C. A. ... ... ... ... ... ... ... ... | 14 Vyvyan Terrace, Clifton. |
| *Mumford, W. G., M.B. Lond. ... ... ... ... | 18 The Circus, Bath. |
| Munden, Charles ... ... ... ... ... ... ... | Ilminster. |
| *Munro, J. M. H., D.Sc. Lond. ... ... ... ... | 12 Grosvenor Place, Bath. |
| *Myles, G T. ... ... ... ... ... ... ... ... | 7 Apsley Road, Clifton. |

| | |
|---|---|
| *Neild, Newman, M.B., B.Ch. Vict. ... ... ... | 9 Richmond Hill, Clifton. |
| *Newman, Charles ... ... ... ... ... ... ... | 156 Cheltenham Road, Bristol. |
| *Newman, Herbert, M.B., B.S.Dur. ... ... | 102 Coronation Road, Bristol. |
| *Newnham, W. H. C., M.B., M.A. Cantab. ... | 3 Lansdown Place, Victoria Square, Clifton. |
| *Nichols, F. C. ... ... ... ... ... ... .. ... | 13 Clarendon Road, Redland. |
| Nicholson, T. D., M.D. Ed. ... ... ... ... | 2 Whiteladies Road, Clifton. |
| *Nixon, J. A., B.A., M.B., B.C. Cantab. ... ... | 18 West Mall, Clifton. |
| Norman, J. H. ... ... ... ... ... ... ... ... | Goodleigh, Winkleigh, Devonshire. |

| | |
|---|---|
| Odell, William, M.D. Durh. ... ... ... ... | Ferndale, Torquay. |
| *Ogilvy, Alexander, M.D., B.Ch., B.A. Dub. | Leny, Clifton Park, Clifton. |
| Openshaw, E. H. ... ... ... ... ... ... ... | Cheddar. |
| Oppenheimer, Son & Co. Ltd. ... ... .. | 179 Queen Victoria Street, E.C. |
| *Ormerod, H. Lawrence, M.B., B.Ch. R.U.I. | 2 Henleaze Road, Westbury Park |

| | |
|---|---|
| Page, G. Shepley ... ... ... ... ... ... ... | 78 Old Market Street, Bristol. |
| Papillon, J. W. ... ... ... ... ... ... ... | Brent Knoll, Somerset. |
| *Parker, George, M.D., M.A. Cantab. ... ... | 14 Pembroke Road, Clifton. |
| Parkinson, Lt. G. S., R.A.M.C. ... ... ... | Military Hospital, Belfast. |
| *Parsons, H. F. ... ... ... ... ... ... ... | The Heath, Stoke Bishop. |
| Parsons, J. H., M.B., B.S., D.Sc. Lond.... ... | 27 Wimpole Street, London, W. |
| Paterson, Donald Rose, M.D., C.M. Ed. ... | 18 Windsor Place, Cardiff. |
| Peake, Arthur W.... ... ... ... ... ... ... | 34 Gloucester Road, Bishopston, Bristol. |
| Peake, G. Arthur ... ... ... ... ... ... | Rodney Place, Cheltenham. |
| Pearse, E. M. ... ... ... ... ... ... ... | 2 Varley Terrace, Liskeard. |
| *Pearse, J., M.D., C.M. Ed. ... ... ... ... ... | 28 St. George's Terrace, Trowbridge. |
| Perdrau, J. A., M.B. Lond. ... ... ... ... | 99 Gower Street, London, W.C. |
| Phillips, C. M., M.D. Brux. ... ... ... ... | Ravenslea, Kensington Hill, Brislington. |
| *Pickering, C. F. ... ... ... ... ... ... ... | 13 Berkeley Square, Bristol. |
| *Pinniger, W. J. H., M.D., B.S. Lond. ... ... | 75 Effingham Road, Bristol. |
| *Porteous, H. B., M.B., Ch.B. Ed. ... ... ... | Newbury House, Weston-super-Mare. |
| Powell, J. J., M.D. Lond. ... ... ... ... ... | 2 Gloucester Road, Bishopston, Bristol. |
| Powell, William, M.B. Lond. ... ... ... ... | Hill Garden, Torquay. |
| Price, D. T., M.B. Lond. ... ... ... ... ... | Castle Cary, Somerset. |

| | |
|---|---|
| *Prichard, Arthur W. ... ... ... ... ... ... ... | 6 Rodney Place, Clifton. |
| *Prowse, Arthur B., M.D. Lond. ... ... ... | 5 Lansdown Place, Clifton. |
| Prowse, W. B.... ... ... ... ... ... ... ... ... | 11 St. George's Place, Brighton. |

| | |
|---|---|
| Randolph, Charles ... ... ... ... ... ... ... | St. Michael's, Milverton, Somerset. |
| *Rattray, J. M., M.D., C.M., M.A. Aberd. ... | Frome. |
| *Rayner, D. C. ... ... ... ... ... ... ... ... ... | 9 Lansdown Place, Clifton, Bristol. |
| Rendall, John ... ... ... ... ... ... ... | Forestside, Lymington. |
| Ritchie, Thomas P. ... ... ... ... ... ... ... | 3 Oakfield Road, Clifton. |
| *Rogers, B. M. H., M.D., B.Ch., B.A. Oxon. ... | 1 Victoria Square, Clifton. |
| *Rolfe, R., B.A., M.B., B.C. Camb. ... ... ... | Park House, Avonmouth. |
| *Rose, F. H. ... ... ... ... ... ... ... ... ... | 13 Westfield Park, Clifton. |
| *Rossiter, George F., M.B. Lond. ... ... | Cairo Lodge, Weston-super-Mare. |
| *Roxburgh, Robert, M.B. Ed. ... ... ... | 1 Victoria Buildings, Weston-super-Mare |
| *Rudge, C. King ... ... ... ... ... ... ... ... | 145 Whiteladies Road, Bristol. |
| *Rudge, F. H. ... ... ... ... ... ... ... ... ... | 145 Whiteladies Road, Bristol. |
| *Rutherford, J. M., M.B., C.M. Edin. ... ... | Brislington House, Bristol. |

| | |
|---|---|
| *Schölberg, H. A., M.B., B.S. Lond. ... ... | University College, Cardiff. |
| Scott, Baty- ... ... ... ... ... ... ... ... | 5 Haverstock Road, Knowle, Bristol. |
| Seward, W. J., M.B. Lond. ... ... ... ... | Colney Hatch Asylum, New Southgate, London, N. |
| *Shaw, J. E., M.B., C.M. Ed. ... ... ... ... | 23 Caledonia Place, Clifton. |
| Sheppard, A. L., M.B., B.S. Dur. ... ... ... | |
| *Sheppard, E. J. .. ... ... ... .. ... ... | 46 St. Paul's Road, Clifton. |
| *Shoolbred, W. A. ... ... ... ... ... ... ... ... | St. Ann's, Chepstow. |
| *Short, A. R., M.D., B.S., B.Sc. Lond. ... ... | 2 Westbourne Place, Queen's Road, Clifton. |
| *Short, L. J., M.B., B.Sc. Lond. ... ... ... | 21 Tyndall Avenue, Clifton. |
| Skelton, Henry, M.D. St. And. ... ... ... | Downend, Gloucestershire. |
| Smith, F. Shingleton, B.A., B.C. Cantab. ... | Grindlay & Co., Bombay. |
| *Smith, G. Cockburn, M.D. Brux. ... ... ... | 14 South Road, Newton Abbot. |
| *Smith, G. Munro ... ... ... ... ... ... ... ... | 18 Apsley Road, Clifton. |
| Smith, L. Shingleton, B.A., M.B. Cantab.... | Honddu House, Brecon. |
| Smith, Rev. Reginald, M.A. Cantab. ... ... | The Vicarage, Walpole St. Andrew, Norfolk. |
| *Smith, R. Shingleton, M.D., B.Sc. Lond. ... | Clifton Park, Clifton. |
| Smith, W., M.B. Lond.... ... ... ... ... ... | 4 Ardgowan Square, Greenock, N.B. |
| *Smith, W. Alexander, M.B., M.A. Oxon. ... | 70 Pembroke Road, Clifton. |

Society, Cardiff Medical (*Hon. Lib.*, Queen Street, Cardiff).

Society, Manchester Medical ... ... ... Medical Society's Library, The Owens College, Manchester.
Society, Plymouth Medical (*Hon. Lib.*, J. Elliott Square).
*Stack, E. H. E., B.A., M.B., B.C. Cantab. ... Arvalee, Clifton Down Road, Clifton.
Staple, J. D. ... ... ... ... ... ... ... ... Clevedon Villa, Cheltenham Road, Bristol.
Statham, R. Whiteside ... ... ... ... ... ... Cheddar, Somersetshire.
*Steele, Charles, M.D. Dur. ... ... ... ... 17 Richmond Hill, Clifton.
Stevens, W. H. ... ... ... ... ... ... ... ... 26 Zetland Road, Bristol.
Stock, P. G. ... ... ... ... ... ... ... ... ... Box 1049, Johannesburg, South Africa.
*Stock, W. S. V., M.B., B.S. Lond. ... ... ... Montrose House, Queen's Road, Clifton
Stoddart, F. Wallis ... ... ... ... ... ... ... Southey House, College Green, Bristol.
*Swain, James, M.D., M.S. Lond. ... ... ... 4 Victoria Square, Clifton.
*Swayne, W. Carless, M.D. Lond. ... ... ... St. Paul's Road, Clifton.
*Symes, J. O., M.D. Lond. ... ... ... ... ... 71 Pembroke Road, Clifton.
Symonds, H. P. ... ... ... ... ... ... ... ... 35 Beaumont Street, Oxford.

Tayler, H. P., M.B., B.C. Cantab. ... ... ... The Abbey House, Bradford-on-Avon.
Taylor, A. L. ... ... ... ... ... ... ... ... ... 22 Downfield Road, Clifton.
Taylor, C. J. ... ... ... ... ... ... ... ... ... 43 Duke Street, Chelmsford.
*Taylor, James ... ... ... ... ... ... ... ... ... 36 Alma Road, Clifton.
*Temple, G. H., M.B., C.M. Ed. ... ... ... Ailanthus, Weston-super-Mare.
Thin, James ... ... ... ... ... ... ... ... ... 54 & 55 South Bridge, Edinburgh.
Thomas, A. Garrod, M.D., C.M. Ed. ... ... Clytha Park, Newport, Mon.
*Thomas, J. D., M.B. Cantab. ... ... ... ... Northwoods, Winterborne,
Thomas, J. Lynn, C.B. ... ... ... ... ... ... 21 Windsor Place, Cardiff.
*Thomas, J. M. M. ... ... ... ... ... ... ... ... 5 Colston Parade, Redcliffe, Bristol.
*Thomas, J. Tubb, D.P.H. .. ... ... ... ... The Halve House, Trowbridge.
*Thomas, R. E., M.B., B.S. Lond. ... ... ... Royal Infirmary, Bristol.
Thomson, Eustace B., M.D., C.M. Glasg. ... Albany Place, Plymouth.
Tivy, W. J. ... ... ... ... ... ... ... ... ... 5 Victoria Square, Clifton.
Tosswill, Leonard R., D.P.H., Cantab. ... Devon County Council Offices, 14 Bedford Circus, Exeter.
Tyley, R. P., M.D. St. And.... ... ... ... ... Wedmore.

Vachell, Herbert R., M.D., C.M. Aberd. ... 18 Newport Road, Cardiff.
*Vickery, W. H. ... ... ... ... ... ,,, ... ... 1 Trewartha Park, Weston-super-Mare.

*Waldo, Henry, M.D., C.M. Aberd. ... ... ... 19 Pembroke Road, Clifton.
*Walker, Cyril H., M.B., B.A. Cantab. ... ... 8 Oakfield Road, Clifton.
Wallace, Rev. Canon, M.A. Oxon. ... ... ... 3 Gloucester Row, Clifton.
*Wallace, John ... ... ... ... ... ... ... ... Lauriston, 15 Knightstone Road, Weston-super-Mare.
Wallace, J. T., M.B., B.Ch., B.A. R.U.I. ... 48 Coronation Road, Bedminster.
*Wallace, James W. ... ... ... ... ... ... ... 1 Greville Road, Southville, Bristol.
*Wallis, G. F. C., M.B., Ch.B. Ed. ... ... ... 10 Westbourne Place, Clifton.
*Walters, C. F. ... ... ... ... ... ... ... ... 19 Whatley Road, Clifton.
*Waterhouse, R., M.D. Lond. ... ... .. ... 28 Gay Street, Bath.
Webber, William W. ... ... ... ... ... ... ... Crewkerne.
*Whicher, A. H. ... ... ... ... ... ... ... ... 115 Queen's Road, Clifton

| | |
|---|---|
| WHITE, C. R. ... ... ... ... ... ... ... ... | Nixon Villa, Merthyr Vale, Glamorgan. |
| WHITE, J. W. ... ... ... ... ... ... ... ... | Orchard House, Nailsea. |
| *WHITE, PERCY W. ... ... ... ... ... ... ... | College Green, Bristol. |
| WIGMORE, J., M.D. Roy. Univ. I. ... ... ... | 20 Green Park, Bath. |
| WILCOX, HENRY, M.B. Aberd. ... ... ... ... | 10 Willington Road, Eastbourne. |
| WILLCOX, R. LEWIS ... ... ... ... ... ... | Warminster. |
| WILLETT, GEORGE G. D. ... ... ... ... ... | Keynsham, Somerset. |
| *WILLIAMS, E. C., M.B., B.C., B.A. Cantab. ... | 159 Whiteladies Road, Clifton. |
| WILLIAMS, H. EGERTON ... ... ... ... ... | The Mount, Newport, Mon. |
| *WILLIAMS, P. WATSON, M.D. Lond.... ... ... | 4 Clifton Park, Clifton. |
| WILLIAMS, S. R., M.B. Lond. ... ... ... ... | Buckfastleigh, Devon. |
| *WILLIAMS, W. ROGER ... ... ... ... ... ... | Morven, Walton-by-Clevedon. |
| WILLIS, W. M.... ... ... ... ... ... ... ... | 7 Regent Street, Nottingham. |
| *WILLS, W. K., M.B., B.C. Camb. ... ... ... | 19 Whiteladies Road, Clifton. |
| *WINDSOR-AUBREY, H. W. ... ... ... ... ... | Coronation Road, Bristol. |
| *WOOD, W. V. ... ... ... ... ... ... ... ... | Glencairn, Wrington. |
| WOOLLCOMBE, WALTER L. ... ... ... ... ... | 16 The Crescent, Plymouth. |
| *WRIGHT, A. J., M.B., B.S. Lond. ... ... ... | 60 St. Paul's Road, Clifton. |
| | |
| YOUNG, J., M.D. Glas. ... ... ... ... ... ... | 14 Chantry Road, Clifton. |

### The Library of the Bristol Medico-Chirurgical Society.

*The following donations have been received since the publication of the List in December.*

*February 28th*, 1910.

| | |
|---|---|
| J. Paul Bush, C.M.G. (1) .. .. .. .. | 1 volume. |
| A. L. Flemming (2) .. .. .. .. .. | 3 volumes. |
| L. M. Griffiths (3) .. .. .. .. .. | 6 volumes. |
| The Medical Officer of the London County Council (4) | 1 volume. |

The Council of the Royal College of Physicians of London (5) .. .. .. .. .. 1 volume.
R. Shingleton Smith, M.D. (6) .. .. .. 14 volumes.
The Surgeon-General of the Public Health and Marine Hospital Service (7) .. .. .. 1 volume.
The Surgeon-General, United States Army (8) .. 1 ,,
Unbound periodicals have been received from Mr. Paul Bush.

### SEVENTY-FIFTH LIST OF BOOKS.

The titles of books mentioned in previous lists are not repeated.

The figures in brackets refer to the figures after the names of the donors, and show by whom the volumes were presented. The books to which no such figures are attached have either been bought from the Library Fund, or received through the *Journal.*

**Albarran, J.** .. .. *Médecine opératoire des Voies urinaires* .. .. 1909
**Andrew, J. G.** .. *Age, Incidence, Sex, and Comparative Frequency in Disease* .. .. .. .. .. .. .. 1909
**Bates, S. H.** .. .. *Open-air Treatment at Home* .. .. .. .. .. 1910
**Bateson, W.** .. .. *Mendel's Principles of Heredity* .. .. (2) 1909
**Bishop, E. S.** .. .. *Lectures on Surgical Nursing* .. .. .. .. .. 1909
**Bosanquet and J. W. H. Eyre, W. C.** *Serums, Vaccines and Toxines* 2nd Ed. 1909
**Bramwell, J. M.** .. *Hypnotism and Treatment by Suggestion* .. 1909
*Catalogue (Index-) of the Library of the Surgeon-General's Office, United States Army.* 2nd Series. Vol. XIV. .. .. (8) 1909
**Clarke, J. J.** .. .. *Congenital Dislocation of the Hip-Joint* .. .. 1910
*Confessio Medici* .. .. .. .. .. .. .. .. .. .. .. .. .. 1909
**Corner, E. M.** .. .. *Male Diseases in General Practice* .. .. .. 1910
**Dubois, P.** .. .. *Pathogeny of the Neurasthenic States* (Trans. by E. G. Richards) .. .. .. .. .. .. 1909
**Eyre, W. C. Bosanquet and J. W. H.** *Serums, Vaccines and Toxines* 2nd Ed. 1909
**Ferrier, D.** .. .. *On Tabes Dorsalis* .. .. .. .. .. .. (6) 1906
**Groves, E. W. H.** .. *A Synopsis of Surgery* .. .. 2nd Ed. 1910
**Hertz, A. F.** .. .. *Constipation and Allied Intestinal Disorders* 1909
**Jellett, H.** .. .. *A Manual of Midwifery* .. .. 2nd Ed. 1910
**Kelynack, T. N.** [Ed.] *Infancy* .. .. .. .. .. .. .. .. .. [1910]
**Keogh (Sir A.),** *et al.* *A Manual of Venereal Diseases* .. .. .. 1907
**Kerr, C. B.** .. .. *Infectious Diseases* .. .. .. .. .. .. .. 1909
**Lake, R.** .. .. .. *Diseases of the Ear* .. .. .. 3rd Ed. 1910
**Lugaro, E.** .. .. *Modern Problems in Psychiatry* (Trans. by D. Orr and R. G. Rows) .. .. .. .. .. 1909
**Mackenzie, J.** .. *Diseases of the Heart* .. .. .. 2nd Ed. 1910
**Moore, N.** .. .. *The Harveian Oration* .. .. .. .. .. (6) 1901
**Muthu, C.** .. .. *Pulmonary Tuberculosis* .. .. .. .. .. .. 1910
**Ormerod, J. A.** .. *On Heredity in Relation to Disease* .. .. (6) 1908

**Payne, J. F.** .. .. *Harvey and Galen* .. .. .. .. .. .. (6) 1897
**Reid, G. A.** .. .. *Alcoholism : A Study in Heredity* .. .. (2) 1901
**Roberts, F. T.** .. *The Harveian Oration,* 1905 .. .. .. (6) 1906
**Robertson, W.** .. *Public Health.* 5 parts .. .. .. .. .. .. [N.D.]
**Russell, P. Sargent and A. E.** *Emergencies of General Practice* .. 1910
**Saleeby, C. W.** .. *Organic Evolution* .. .. .. .. .. .. (2) [N.D.]
**Salisbury, J. H.** .. *The Relation of Alimentation and Disease* (6) 1888
**Sargent and A. E. Russell, P.** *Emergencies in General Practice* .. 1910
**Savage, G. H.** .. *On Experimental Psychology and Hypnotism* (6) 1909
*Secret Remedies* .. .. .. .. .. .. .. .. .. .. .. .. .. 1909
**Smith, E.** .. .. *Some Common Remedies* .. .. .. .. .. 1910
**Taylor, F.** .. .. *The Need of Research in Medicine* .. .. (6) 1908
**Tunstall, F. J. Warwick and A. C.** *First Aid to the Injured and Sick* 6th Ed. 1910
**Walford, W. G.** .. *Cerebral Congestion and Tight Neck Clothing* 1910
**Walley,** .. .. *Meat Inspection* 5th Edition by S. Stockman 1909
**Walsh, D.** .. .. *Quack Remedies* .. .. .. .. .. .. .. .. 1909
**Warwick and A. C. Tunstall, F. J.** *First Aid to the Injured and Sick* 6th Ed. 1910
**Williams, J.** .. .. *Roller Skating for Health and Pleasure* .. .. 1910
**Williams, P. W.** .. *Rhinology* .. .. .. .. .. .. .. .. .. .. 1910

### TRANSACTIONS, REPORTS, JOURNALS, &c.

American Association of Genito-Urinary Surgeons, Transactions of the .. .. .. .. .. .. .. .. .. .. Vols. III, IV. 1908–09
American Laryngological Association, Transactions of the .. .. 1909
American Surgical Association, Transactions of the .. Vol. XXVII 1909
Archiv für Gynaekologie .. .. Bds. LXXXVII, LXXXVIII 1909
Archiv für klinische Chirurgie .. Bds. LXXXVIII, LXXXIX 1909
Army Medical Department, Report for 1908 .. (1) Vol. L 1909
Association of American Physicians, Transactions of the Vol. XXIV 1909
Beiträge zur klinischen Chirurgie .. .. .. .. .. Bds. LXI–LXIII 1909
Bristol Directory, Wright's .. .. .. .. .. .. .. .. .. .. .. .. 1910
British Medical Journal, The .. .. .. .. .. .. .. .. Vol. II for 1909
Catalogue of Books, The English, for 1908 .. .. .. .. .. .. .. 1909
Clinical Journal, The .. .. .. .. .. .. .. .. Vol. XXXIV 1909
Deutsche Zeitschrift für Chirurgie .. .. .. .. Bds. XCVIII, C 1909
Hazell's Annual .. .. .. .. .. .. .. .. .. .. .. .. .. .. .. .. 1910
Henry Phipps Institute, Fifth Annual Report of the .. .. .. .. .. 1909
Hospitals—Guy's Hospital Reports .. .. .. .. .. Vol. LXIII 1909
Lancet, The .. .. .. .. .. .. .. .. .. .. .. .. .. Vol. II for 1909
London County Council, Report of the Medical Officer for 1908 (4) 1909
Medical Directory, The .. .. .. .. .. .. .. .. .. .. .. .. 1910
Parasitology .. .. .. .. .. .. .. .. .. .. .. .. .. .. .. Vol. I 1908
Philippine Journal of Science, The .. .. .. .. .. Vol. III 1908
Public Health Reports issued by the Surgeon-General, Public Health and Marine Hospital Service. (7) Vol. XXIII, Part 2 1909

| | | |
|---|---|---|
| Revue de Chirurgie .. .. .. .. .. .. .. .. | Tom. XXXIX | 1909 |
| Royal College of Physicians of London, List of Fellows, etc., of the | (5) | 1910 |
| Royal Society of Medicine, Proceedings of the .. .. .. .. | Vol. II | 1909 |
| Therapeutic Gazette, The .. .. .. .. .. .. | Vol. XXXII | 1908 |
| Whitaker's Almanack .. .. .. .. .. .. .. .. .. .. .. .. .. .. | | 1910 |
| Year-Book of Scientific and Learned Societies .. .. .. .. .. .. | | 1909 |

### MEETINGS OF SOCIETIES.

### Bristol Medico-Chirurgical Society.

### Local Medical Notes.

**University of Bristol.**—Faculty of Medicine:—

EXAMINATION SUCCESSES.

London University.—*First Examination for Medical Degrees:* H. J. McCurrich, H. J. D. Smythe.

*M.D.* (*Medicine*): J. F. Blackett.

Conjoint Board.—*Materia Medica:* R. S. S. Statham. *Anatomy and Physiology:* W. Worger. *Medicine:* B. C. Eskell, H. R. B. Hull, R. S. S. Statham. *Surgery:* S. H. Kingston.* *Midwifery:* V. Pinnock, C. H. Hart, W. A. Reynolds.

F.R.C.S.Ireland.—M. G. Dobbyn.

APPOINTMENTS.

Boulter, W. E., L.D.S.Eng.—Honorary Dental Surgeon to the Queen Victoria Jubilee Convalescent Home.

Aubrey, T., M.B.Lond.—Medical Officer of Health for the Warmley District Council.

Pinniger, W. J. H., M.B., B.S.Lond.—Medical Officer to Müller's Orphan Houses, Bristol.

Annan, John Laing, M.B., Ch.B.Edin.—Demonstrator of Anatomy.

Burridge, William, B.A.Oxon.—Demonstrator of Physiology.

### BRISTOL.

**University of Bristol.**—*Ordinances and Regulations.*—Some of our readers may not be aware that provisional ordinances and regulations for degrees, certificates and diplomas have been issued, and can be obtained from the Registrar. It will be noted that allowances are made for old Bristol students who are already qualified.

**Bristol Eye Hospital.**—The annual meeting of this institution was held on February 3rd, and presided over by the Lord Mayor, who, in commenting upon the excellent report, called attention to the number of patients who resided in outlying towns and villages, and he thought the Hospital Sunday Fund should benefit more from these places. During 1909 there were 441 in-patients and 8,628 out-patients. There was a balance of £1,100 due to the treasurer. Attention was called to the Fête which was to be held in the Zoological Gardens in the summer, in aid of the Eye Hospital.

**Queen Victoria Jubilee Convalescent Home.**—At the annual meeting, held recently, the medical report stated that during 1909 1,847 patients had been admitted, 1,016 males and 831 females; of this number the Royal Infirmary had sent 611 and the General Hospital 652; 334 were free patients, 148 paying patients, 68 from the Bristol Dispensary, 17 from the Clifton Dispensary, and 17 from the Eye Hospital. The average daily number was 79, and the average stay just over 15 days. The total income for the year amounted to £3,174, and the expenditure to £2,839.

* Completes examination.

**Bristol Dispensary.**—During 1909 10,993 patients were treated at this institution, and 687 midwifery notes were issued, an increase of 151 over the preceding year. These notes guarantee the provision of trained midwives, and of medical aid when required. The annual expenditure exceeded the income by £22, but there was a balance in hand of £130.

**Clifton Dispensary.**—The ninety-seventh annual report of this useful institution stated that 2,327 patients had been treated during 1909, including 74 midwifery cases. At the close of the year a small unfavourable balance remained.

### BATH.

**Royal United Hospital.**—The annual report of this institution shows that during 1909 there were 1,385 in-patients as against 1,298 in the preceding year, and 14,155 out-patients were treated, compared with 13,765 in 1908. 279 patients were visited at their homes. At the close of the year there was a deficit of £634.

### PLYMOUTH.

At the annual meeting of the **Plymouth Ear and Throat Hospital,** held recently, the medical report showed that during 1909 1,013 patients had been treated. A favourable balance was shown by the financial statement. It was resolved to adopt the suggestion of the Committee, and to proceed with a scheme for rebuilding the institution. The first section of the scheme is estimated to cost about £1,000.

**Royal Eye Infirmary.**—During 1909 the out-patients attending this institution numbered 2,372 (an increase of 128 over 1908), and 300 in-patients had been admitted, as against 278 in the preceding year. The daily average of in-patients was 24. The financial statement showed that with an income of £1,845 a favourable balance remained.

### WESTON-SUPER-MARE.

At a special meeting of the Committee of Management of the **Royal West of England Sanatorium,** held at the end of 1909, it was decided to admit no more patients suffering from pulmonary tuberculosis. The institution was closed during November and December, and thoroughly disinfected and cleansed, at a cost of £400, and in January the institution was reopened for the admission of patients.

[illegible] **Health Report.**—The Medical Officer of Health, in his report for 1909, estimates the population at 23,500; the death-rate was 11.74 per 1,000. Exclusive of visitors, the death-rate was 10.6 per 1,000.

